# Matured Hop-Derived Bitter Components in Beer Improve Hippocampus-Dependent Memory Through Activation of the Vagus Nerve

**DOI:** 10.1038/s41598-018-33866-1

**Published:** 2018-10-18

**Authors:** Tatsuhiro Ayabe, Rena Ohya, Yoshimasa Taniguchi, Kazutoshi Shindo, Keiji Kondo, Yasuhisa Ano

**Affiliations:** 1 Research Laboratories for Health Science & Food Technologies, Kirin Company Ltd, Yokohama, Kanagawa 236-0004 Japan; 2 Central Laboratories for Key Technologies, Kirin Company Ltd, Yokohama, Kanagawa 236-0004 Japan; 3 Department of Food and Nutrition, Japan Women’s University, Tokyo, 112-8681 Japan

## Abstract

Improving and maintaining memory function is effective in preventing cognitive decline and dementia. Previously, we demonstrated that iso-α-acids, the hop-derived bitter components in beer, prevent cognitive impairment in an Alzheimer’s disease mouse model. In this report, we investigated the effects of matured hop bitter acids (MHBA) containing components of oxides derived from α- and β-acids, and structurally similar to iso-α-acids, on cognitive function using behavioral pharmacological procedures. MHBA and the representative components of MHBA, 4′-hydroxyallohumulinone (HAH) and 4′-hydroxy-*cis*-alloisohumulone (HAIH) improved spatial working memory in scopolamine-induced amnesia mice. MHBA also enhanced episodic memory in the novel object recognition test (NORT). The administration of MHBA increased the amount of norepinephrine (NE) and NE release into cerebrospinal fluid (CSF) in hippocampus. The MHBA activity in improving memory function was attenuated by treatment with a β-adrenergic receptor inhibitor. In addition, vagotomized mice did not display the memory improvement induced by MHBA. Together, our results suggest that MHBA improves memory function via stimulation of the vagus nerve and enhancement of NE release in the hippocampus. Vagus nerve activation by the intake of food materials including MHBA may be a safe and effective approach for improving cognitive function.

## Introduction

Memory plays an essential role in daily life. However, some memory functions peak at the young adult age and decrease slightly throughout life span^1,2^. Thus, improving and maintaining memory function through daily exercise or dietary habits beginning at a younger age may be effective in preventing cognitive decline or dementia. Results of a meta-analysis of the relationship between alcoholic beverage consumption and cognitive risk have suggested that the daily intake of a low to moderate amount of alcoholic beverage may reduce the risk for dementia^3^. While this finding may depend on the effects of alcohol itself, specific compounds contained in alcoholic beverages may be involved. Resveratrol, a polyphenolic compound found in red wine, has been reported to improve cognitive function in young adult mice^4^, several murine dementia models^5,6^ and healthy human adults^7,8^. Our group has focused on the constituents of beer, and we found that iso-α-acids, major bitter components in beer derived from hops (*Humulus lupulus* L.), improve cognitive impairment in an Alzheimer’s disease (AD) mouse model^9^ and high fat diet-induced obese mice^10^. Xanthohumol, a prenylated flavonoid derived from hops, and its derivatives have also been reported to exhibit cognitive improvement in mice^11,12^. However, there remains a number of hop-derived components in beer that have not been investigated for their effects on cognitive function.

Hops contain α- and β-acids, and α-acids isomerize to iso-α-acids during the brewing process. Iso-α-acids have been studied widely and are reported to exhibit various physiological benefits, not only on cognitive function. Our group previously demonstrated that iso-α-acids prevent lipid accumulation and insulin resistance in diet-induced obese rodents^13–15^ and improve glucose metabolism and body fat accumulation in a clinical trial^16^.

The α- and β-acids are also converted to oxidized components called matured hop bitter acids (MHBA) that contain a β-tricarbonyl formula and a common chemical structure with iso-α-acids^17–19^. Because of their structural similarity to iso-α-acids, MHBA are expected to have similar physiological effects as those of iso-α-acids, such as preventing obesity and cognitive impairment. Our group previously demonstrated that diet supplementation of MHBA reduces body weight gain and epididymal fat accumulation in high fat diet-fed rodents, and that oral administration of MHBA promotes thermogenesis in brown adipose tissues (BAT) by elevating sympathetic nerve activity^20^. The BAT stimulation is blocked by vagotomy operation, suggesting that the effect of MHBA is mediated through activation of the vagus nerve. Further, continuous ingestion of MHBA reduced body fat in a clinical trial^21^. On the other hand, the effects of MHBA on cognitive functions have not been investigated.

The vagus nerve mediates visceral stimuli to the locus coeruleus (LC), and these signals are communicated to various regions of the brain through noradrenergic nerves, which release mainly norepinephrine (NE) as a neurotransmitter^22^. NE plays important roles in several mood disorders and epilepsy^23,24^, and vagus nerve stimulation has been studied as a therapeutic strategy for these diseases^25–27^. Recently, NE was reported to be involved in AD and that vagus nerve stimulation may relieve the symptoms of AD^28^. Thus, MHBA may improve cognitive function through vagus nerve stimulation.

In the present study, we investigated the effects of MHBA on cognitive function in young normal adult mice and scopolamine-induced amnesia model mice, using behavioral pharmacological methods. Scopolamine is a muscarinic antagonist that impairs learning and memory function temporarily by inhibiting cholinergic neuronal systems. Thus, scopolamine-induced amnesia models are used widely to screen drugs for effects on dementia and cognitive decline^29^. In addition, we examined the mechanisms underlying the function of MHBA, focusing on the vagus nerve and adrenergic signaling.

## Results

### MHBA improves spatial working memory in a scopolamine-induced amnesia mouse model

**Figure 1:**
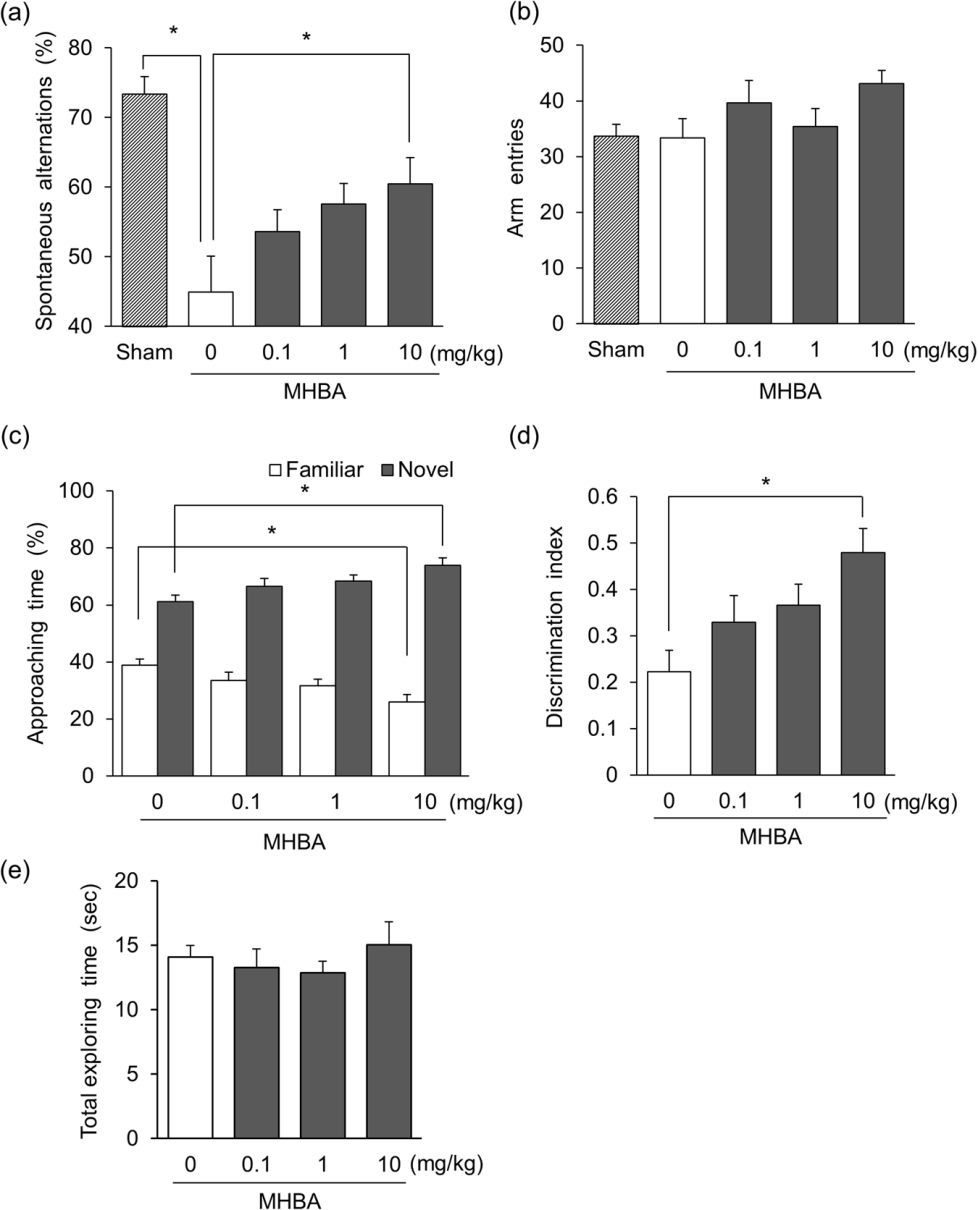
MHBA improve spatial working memory and episodic memory. (**a**,**b**) Spatial working memory after MHBA administration was evaluated by spontaneous alternation in the Y-maze test using scopolamine-induced amnesia model mice. Mice were administered MHBA (0, 0.1, 1, 10 mg/kg) orally and intraperitoneally injected with scopolamine (0.8 mg/kg) after 40 min. For the sham group, distilled water and saline were used instead of MHBA and scopolamine, respectively. Sixty min after the oral administration, spontaneous alternation behavior (**a**) and the number of arm entries (**b**) in the Y-maze was observed for 8 min (**c**–**e**). Episodic memory was evaluated in the novel object recognition test (NORT). Mice were administered MHBA (0, 0.1, 1, 10 mg/kg) orally. The time spent approaching novel and familiar objects during the recall period was measured (**c**). The discrimination index was calculated as the following formula: (novel object exploration time minus familiar object exploration time)/(total exploration time) (**d**). The total time spent exploring both objects was calculated (**e**). All values are expressed as means ± SEM (n = 8–10 mice per group). *p < 0.05 versus each group.

The scopolamine-induced amnesia mouse model has been used widely to screen drugs for an effect on dementia and cognitive decline^30,31^. To investigate the effects of MHBA on spatial working memory, we performed the Y-maze test using scopolamine-induced amnesic mice. Spontaneous alternation of scopolamine-treated mice was significantly decreased when compared with saline-treated sham mice, indicating that amnesia was induced successfully. MHBA administration at a dose of 10 mg/kg significantly increased spontaneous alternation compared with control mice [F(4,35) = 8.35, p < 0.001] (Fig. 1a). Since there was no difference in the number of arm entries between each group [F(4,35) = 1.83, p = 0.145] (Fig. 1b), MHBA dose-dependently enhanced spatial working memory in the amnesia model mice. Several acetylcholinesterase (AChE) inhibitors have been reported to enhance spontaneous alternation in scopolamine-induced amnesia^31,32^, but MHBA exhibited no effects on the AChE activity (data not shown).

### MHBA enhances episodic memory in novel object recognition

Next, we conducted the novel object recognition test (NORT) using normal mice to evaluate the effects of MHBA on episodic memory. Memory performance in this test declines dependent on the delay between the two trials^33^. Thus, improvement in NORT performance suggests an enhancement of memory acquisition and retention. The 24 h-delay method in this study has been used to evaluate the effects of drugs or food-derived components^34,35^. MHBA at a dose of 10 mg/kg significantly increased the proportion of time spent approaching the novel object over that of control mice [F(3,36) = 4.67, p = 0.007] (Fig. 1c). The discrimination index, which indicates the tendency to approach the novel object, was also significantly increased in the 10 mg/kg MHBA administered group compared with the control group [F(3,36) = 4.67, p = 0.007] (Fig. 1d). There was no difference in the total time spent exploring the both objects [F(3,36) = 0.43, p = 0.735] (Fig. 1e), indicating that MHBA enhanced episodic memory in normal mice.

### Representative components contained in MHBA improve spatial working memory

**Figure 2:**
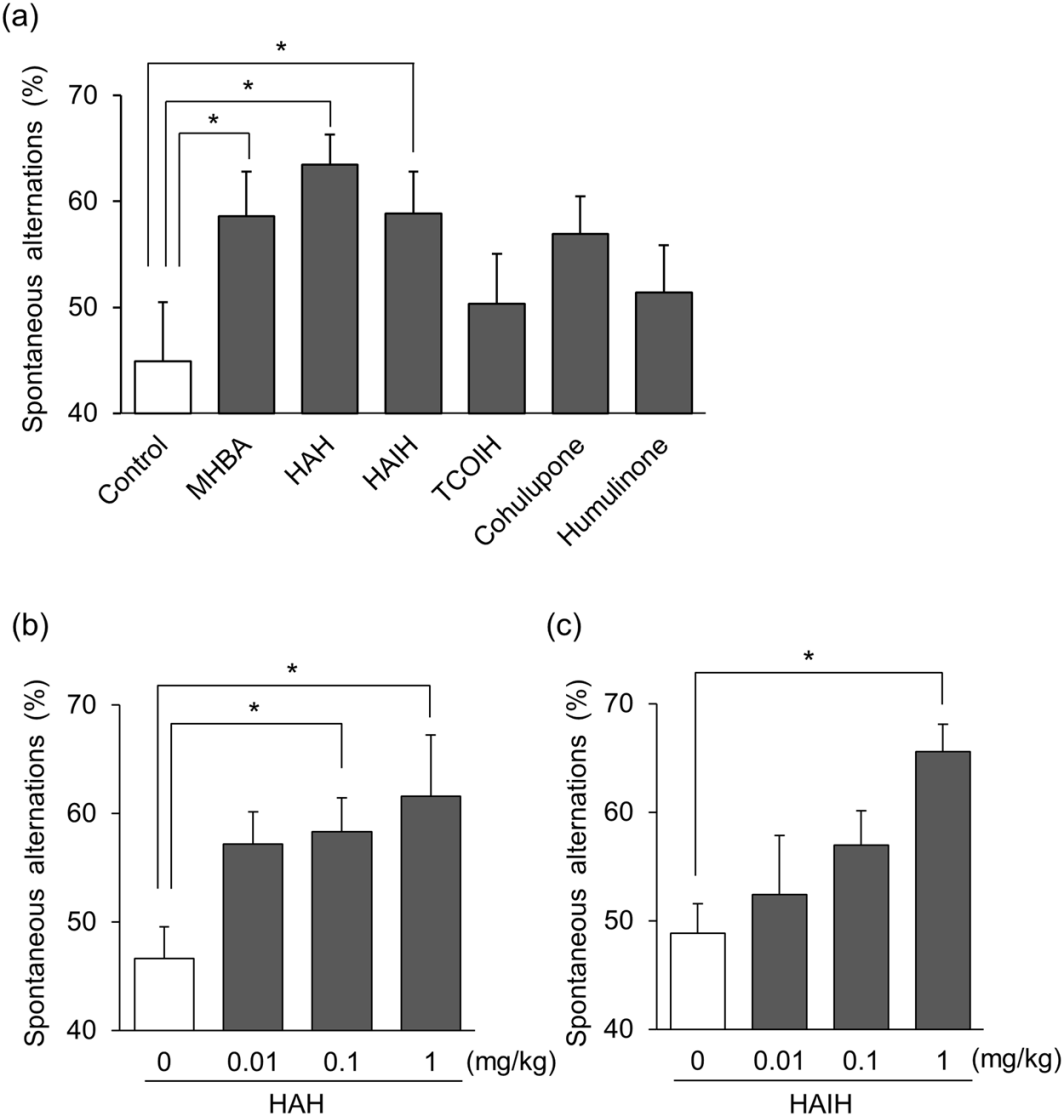
Representative compounds in MHBA improve spatial working memory. The effect of each compound contained in MHBA on spatial working memory was evaluated in the Y-maze test (**a**). Mice were administered MHBA orally at a dose of 10 mg/kg. The component compounds, 4′-hydroxyallohumulinones (HAH), 4′-hydroxyalloisohumulones (HAIH), tricyclooxyisohumulones A (TCOIH-A), hulupones, and humulinones were administered orally at 1 mg/kg, respectively. (**b**,**c**) Mice were administered HAH (0, 0.01, 0.1, 1 mg/kg) (**b**) or HAIH (0, 0.01, 0.1, 1 mg/kg) (**c**) orally. Scopolamine (0.8 mg/kg) was administered 40 min later, and the Y-maze test was conducted at 60 min after the oral administration. Spontaneous alternation behavior was observed for 8 min. All values are expressed as means ± SEM (n = 8–10 mice per group). *p < 0.05 versus control (0 mg/kg) group.

MHBA are a mixture of α- and β-acid oxides with β-tricarbonyl structures^17–19^. Five compounds (4′-hydroxyallohumulinone, HAH; 4′-hydroxy-*cis*-alloisohumulone, HAIH; tricyclooxyisohumulone A, TCOIH-A; cohulupone; and humulinone) found in MHBA were obtained, and the effect of each compound was examined in the Y-maze test using amnesia model mice. HAH and HAIH at a dose of 1 mg/kg significantly increased spontaneous alternation respectively, and the effects were comparable to those of MHBA at a dose of 10 mg/kg [F(3,36) = 4.67, p = 0.007] (Fig. 2a). The effects of HAH and HAIH were investigated at different doses, and HAH and HAIH significantly enhanced spontaneous alternation in a dose-dependent manner [F(3,34) = 3.20, p = 0.036; F(3,36) = 3.96, p = 0.016] (Fig. 2b,c). Thus, components in MHBA that improve spatial working memory were identified.

### MHBA increases the levels of NE in the hippocampus

**Figure 3:**
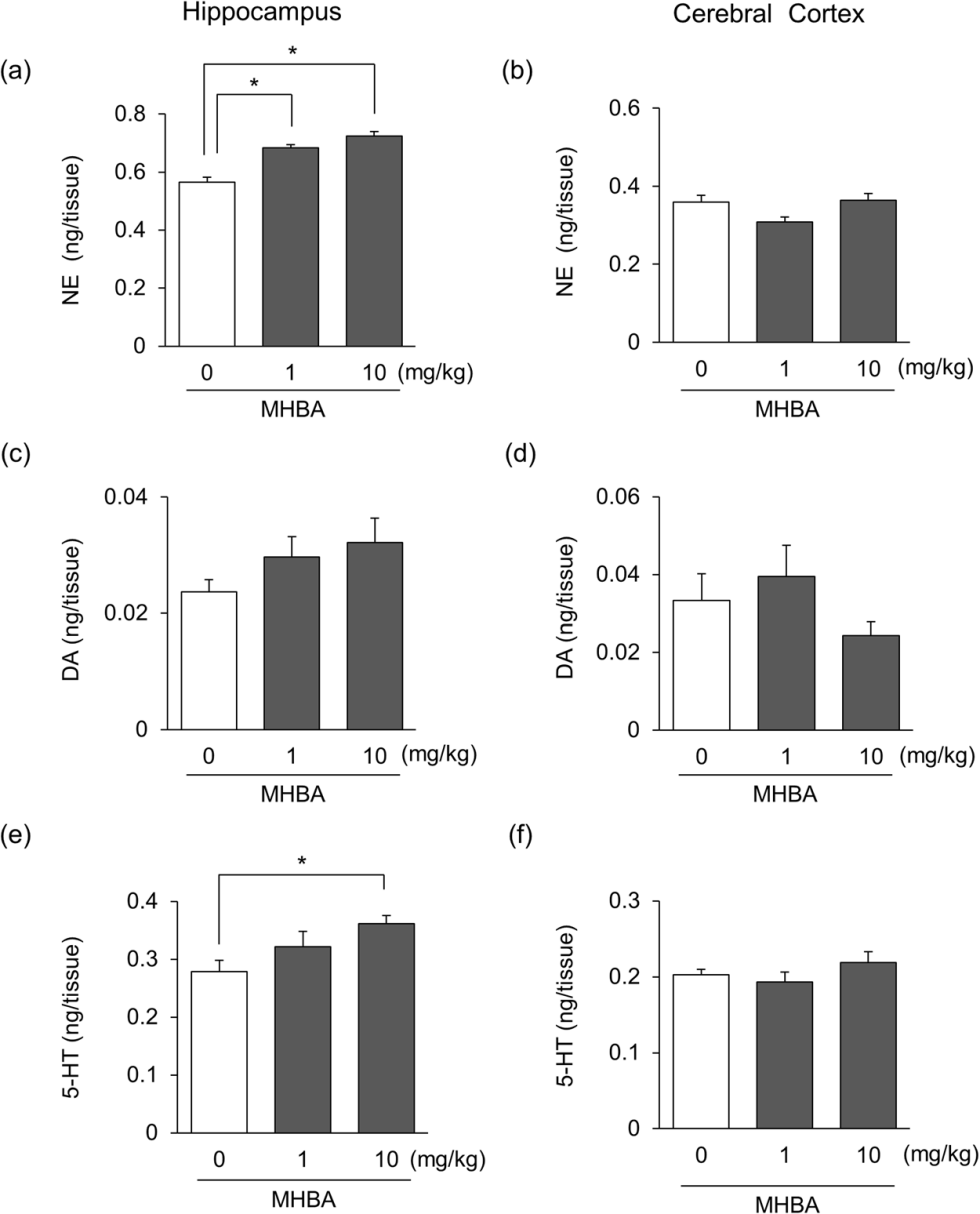
Repeated administration of MHBA increases hippocampal levels of monoamines. Mice were administered MHBA (0, 1, 10 mg/kg) orally for 5 days. The levels of norepinephrine (**a**,**b**), dopamine (**c**,**d**) and serotonin (**e**,**f**) in the hippocampus and cerebral cortex were determined at 60 min after the last administration, respectively, using HPLC-ECD. All values are expressed as means ± SEM (n = 10 mice per group). *p < 0.05 versus control (0 mg/kg) group.

Stimulation of the vagus nerve has been reported to increase the levels of brain monoamines, especially norepinephrine (NE)^22^. Since our group reported previously that MHBA stimulates vagal afferents, we administered MHBA for 5 days and measured the levels of brain monoamines. In the hippocampus, the levels of NE were elevated significantly by MHBA administration at doses of 1–10 mg/kg [F(2,27) = 4.95, p = 0.015], and the levels of serotonin (5-HT) were also increased by MHBA at a dose of 10 mg/kg [F(2,27) = 3.96, p = 0.031], dose-dependently (Fig. 3a,e). The levels of dopamine (DA) in hippocampus were increased dose-dependently, but the differences were not significant [F(2,27) = 1.67, p = 0.207] (Fig. 3c). On the other hand, the increases of NE [F(2,27) = 3.70, p = 0.038], DA [F(2,27) = 1.40, p = 0.264], and 5-HT [F(2,27) = 1.14, p = 0.336] were not observed in the cerebral cortex (Fig. 3b,d,f). The continuous administration of MHBA elevated hippocampal levels of monoamines, especially NE.

### A single administration of MHBA enhances hippocampal NE release

**Figure 4:**
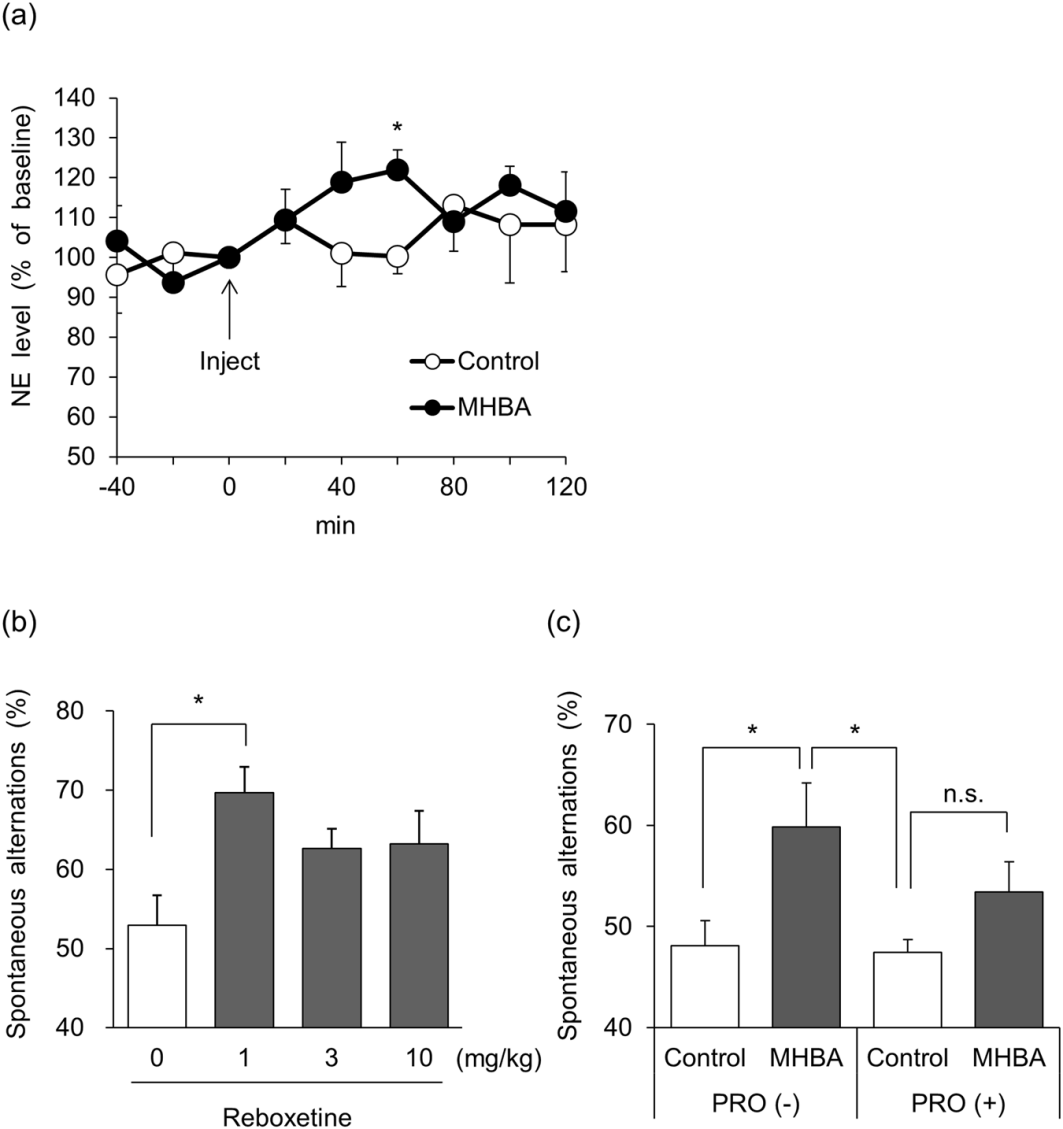
A single administration of MHBA increases hippocampal NE release. (**a**) CSF norepinephrine levels in the hippocampus were measured using the microdialysis system. The dialysis probe was inserted into the hippocampal region (bregma −3.5 mm anterior, −3.0 mm lateral, and 1.8 mm vertical). Mice were administered MHBA (0, 10 mg/kg) orally. CSF was collected every 20 min before and after the MHBA administration. NE concentration in the CSF sample was determined using HPLC-ECD. (**b**) The effect of reboxetine on spatial working memory was evaluated in the Y-maze test. Mice were administered reboxetine (0, 1, 3, 10 mg/kg) orally and were intraperitoneally-injected scopolamine (0.8 mg/kg) at 40 min after reboxetine administration. At 60 min after sample administration, spontaneous alternation behavior was observed for 8 min. (**c**) The effect of MHBA on spatial working memory was examined using scopolamine-induced amnesia model mice with a β-adrenergic receptor blocker. Mice were administered MHBA (0, 10 mg/kg) orally and intraperitoneally-injected scopolamine (0.8 mg/kg) at 40 min thereafter. Propranolol (10 mg/kg) or saline was intraperitoneally-injected at the same time with scopolamine injection. Sixty min after the sample administration, spontaneous alternation behavior was observed for 8 min. All values are expressed as means ± SEM (n = 8–10 mice per group). *p < 0.05 versus control (0 mg/kg) group.

We examined the effect of a single oral administration of MHBA on hippocampal NE using the microdialysis system. A single administration of MHBA (10 mg/kg) significantly increased the NE concentration in cerebrospinal fluid time-dependently, compared with control mice at 60 min after administration [t(12) = 3.26, p = 0.007] (Fig. 4a). It is noteworthy that MHBA enhanced hippocampal NE release at the same time point at which the mice exhibited memory improvement in behavioral pharmacological tests.

### The β-adrenergic receptor is involved in the effect of MHBA on memory function

Since MHBA enhanced hippocampal NE release, the elevation of NE in the hippocampus may improve hippocampus-dependent memory functions. Prior to the evaluation using MHBA, we examined the effect of reboxetine, a NE reuptake inhibitor, using the Y-maze test. Reboxetine at a dose of 1 mg/kg significantly elevated spontaneous alternation compared with the control group [F(3,28) = 3.90, p = 0.019] (Fig. 4b). This result suggests that the increase of NE levels improves spatial working memory. We examined the involvement of NE in the activity of MHBA by inhibiting β-adrenergic receptor (β-AR), which is the main receptor of NE. Propranolol (PRO), a potent antagonist of β-AR, was administered to scopolamine-induced amnesia mice, and the effect of MHBA was evaluated in the Y-maze test. MHBA (10 mg/kg) significantly enhanced spontaneous alternation in mice treated with scopolamine, but not PRO, when compared with vehicle-treated mice. On the other hand, MHBA (10 mg/kg) did not change spontaneous alternation in mice treated with scopolamine and PRO, compared with vehicle-treated mice [F(3,36) = 3.70, p = 0.020] (Fig. 4c). These results indicate that β-AR is involved in the memory improvement activity of MHBA, suggesting that NE signaling contributes to the improvement of cognitive function by MHBA administration.

### The effect of MHBA is mediated by vagus nerve stimulation

**Figure 5:**
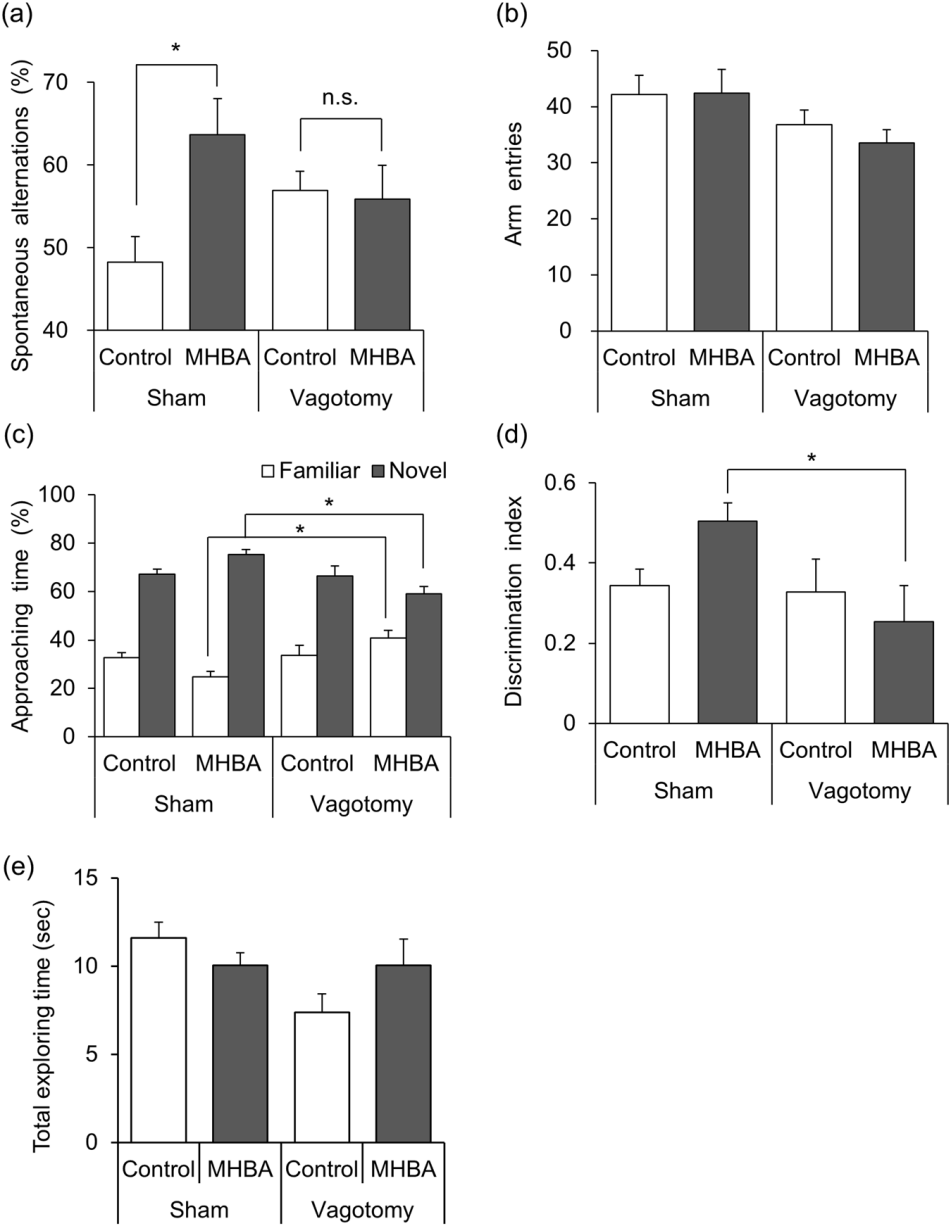
The effect of MHBA is mediated by vagus nerve stimulation. Vagotomy was performed at 5 weeks of age and used for the experiments at 6–8 weeks of age. (**a**,**b**) Spatial working memory after MHBA administration was evaluated by spontaneous alternation in the Y-maze test using scopolamine-induced amnesia model mice. Mice were administered MHBA (0, 10 mg.kg) orally and intraperitoneally injected scopolamine (0.8 mg/kg) after 40 min. 60 min after the oral administration, spontaneous alternation behavior (**a**) and number of arm entries (**b**) in the Y-maze apparatus was observed for 8 min. (**c**–**e**) Percentage of time approaching to each object and discrimination index was evaluated in novel object recognition test. Mice were administered MHBA (0, 10 mg/kg) orally. The time spent approaching to novel and familiar object in recall period was measured (**c**). Discrimination index was calculated using the following formula: (novel object exploration time minus familiar object exploration time)/(total exploration time) (**d**). The total time spent exploring both objects was calculated (**e**). *p < 0.05 versus each group.

In order to elucidate the involvement of the vagus nerve in the activity of MHBA, we performed the Y-maze test and NORT using vagotomized mice. MHBA (10 mg/kg) significantly increased spontaneous alternation during the Y-maze test in sham mice. On the other hand, in vagotomized mice, MHBA did not change spontaneous alternation during the Y-maze test [F(3,35) = 3.14, p = 0.037] (Fig. 5a). In NORT, the proportion of time approaching the novel object and the discrimination index in MHBA-treated vagotomized mice significantly decreased compared with MHBA-treated sham mice [F(3,32) = 5.36, p = 0.004], suggesting that the vagotomy operation diminished the cognitive function of MHBA-administered mice (Fig. 5c,d). The vagotomy operation and MHBA treatment did not change the total time spent exploring both objects [F(3,32) = 2.78, p = 0.057] (Fig. 5e). These results suggest that the effects of MHBA on improving memory function were mediated by stimulation of the vagus nerve. The completion of vagotomy was assessed by evaluating the satiety effect of cholecystokinin-octapeptide (CCK-8), which was reported previously to act via the vagus nerve^36–38^. The administration of CCK-8 reduced food intake in sham mice, but not in vagotomized mice, indicating that the mice used in this experiment were vagotomized successfully (Supplementary Fig. 1).

## Discussion

In the present study, we identified a novel activity of MHBA in improving spatial working memory and episodic memory. In addition, these effects were mediated by stimulation of the vagus nerve. Several studies have reported that stimulating the vagus nerve electrophysiologically or enterobacterially influences emotional behaviors^39,40^ and cognitive functions^41–43^. Thus, the vagus nerve is considered to be a therapeutic target for several central nervous system (CNS) disorders including dementia and cognitive decline^28^. Results of the present study suggest that stimulation of the vagus nerve by food materials may be an effective and safe approach for treating cognitive impairment or dementia, because they influence brain function without penetrating the blood-brain barrier.

Our results showed that MHBA activates the vagus nerve and increases hippocampal NE release. Since spontaneous alternation behavior and novel object recognition are reported to be dependent on hippocampal activity^44^, an increase in hippocampal NE levels may be involved in the hippocampal memory improvement activity of MHBA. Afferent vagal nerve projections to the locus coeruleus (LC), the main source of NE in the brain, activates noradrenergic signals to various regions of the brain including the hippocampus^22,45–47^, which is consistent with our present observations. Hippocampal NE is essential for contextual memory and episodic memory^48–50^. In the present study, we observed that an elevation of NE concentration by inhibiting the NE reuptake transporter improves spontaneous alternation in the Y-maze test, suggesting that NE also enhances spatial working memory.

MHBA are comprised of α- and β-acid oxides, which have a common β-tricarbonyl moiety^17,18^. We demonstrated that 4′-hydroxyallohumulinone (HAH) and 4′-hydroxyallo-*cis*-isohumulone (HAIH), representative components of MHBA, improve cognitive function significantly. The common β-tricarbonyl structure among HAH, HAIH, and iso-α-acids likely contribute to their potent activity (Supplementary Fig. 2). Previously, we demonstrated that the dietary supplementation of iso-α-acids prevents cognitive impairment in Alzheimer’s disease (AD) model mice^9^. MHBA also improved working memory in amyloid β oligomer intraventricular-injected amnesia model mice (Supplementary Fig. 3), preliminarily suggesting their effects on AD.

In conclusion, results of the present study reveal that the administration of MHBA improves hippocampus-dependent memory, including spatial working memory, in scopolamine-induced amnesia model mice and episodic memory in normal mice. MHBA increased NE levels and NE release into the CSF in the hippocampus. The effect of MHBA on memory improvement was blocked by β-AR inhibition and vagus nerve depletion. Taken together, our results suggest that MHBA improves memory function via stimulation of the vagus nerve and subsequent hippocampal NE release. Vagus nerve stimulation by food materials may be effective in improving memory function.

## Materials and Methods

### Materials

MHBA and each representative component (4′-hydroxyallohumulinone, HAH; 4′-hydroxy-*cis*-alloisohumulone, HAIH; tricyclooxyisohumulone A, TCOIH-A; cohulupone; and humulinone) were prepared from hop pellets or synthesized as described previously^17–19,51^. 4′-Hydroxy-*cis*-alloisohumulone (HAIH) was purified from MHBA as described in the supplementary information. Norepinephrine (NE), dopamine (DA), serotonin (5-HT), scopolamine, reboxetine, and propranolol were purchased from Sigma Aldrich Co. (St. Louis, MO, USA).

### Animals

Five-week-old male ICR mice and vagotomized male ICR mice were purchased from Charles River Japan Inc. (Tokyo, Japan). Vagotomy was performed in 5 week old ICR in the laboratory of Charles River Japan Inc. and were used for experiments after the mice were 6 weeks old. Mice were maintained at room temperature (23 ± 1 °C) under constant 12-h light/dark cycles (light period from 8:00 am to 8:00 pm). All mice were acclimatized by feeding a standard rodent diet, CE-2 (Clea Japan, Tokyo, Japan), for 7 days. All animal care and experimental procedures were in accordance with the guidelines of the Animal Experiment Committee of Kirin Company Ltd., and all efforts were made to minimize suffering. All studies were approved by the Animal Experiment Committee of Kirin Company Ltd., and the approval IDs were AN10174-Z00, AN10213-Z00, AN10237-Z00, AN10268-Z00, AN10316-Z00, AN10349-Z00, AN10474-Z00, AN10506-Z00, and AN10519-Z00.

### Brain sample preparation and analysis of monoamines

Mice were administered MHBA (0, 1, 10 mg/kg) orally for 5 days. Sixty min after the last administration, mice were sacrificed, and the cerebral cortex and hippocampus were collected. Samples were homogenized using a multi-beads shocker (Yasui Kikai, Osaka, Japan) in TBS buffer containing a protease inhibitor cocktail (BioVision, Mountain View, USA). The homogenates were centrifuged at 50,000 g for 30 min, and the supernatants were collected. Samples were deproteinized by adding 0.2 M perchloric acid. Monoamines were quantified using high-performance liquid chromatography coupled with electrochemical detection (HPLC-ECD; Eicom, Kyoto, Japan). Monoamines were separated using SC-5ODS columns (Eicom) and the amounts of norepinephrine (NE), dopamine (DA), and serotonin (5-HT) were determined.

### Microdialysis

Mice were anesthetized using pentobarbital (Somnopentyl; Kyoritsu, Tokyo, Japan). Lidocaine (Xylocaine Jelly; AstraZeneca, London, UK) was used for calvarial local anesthesia. Each mouse was placed in a stereotaxic frame (Narishige, Tokyo, Japan). A guide cannula (Eicom) was inserted into the hippocampal region (bregma −3.5 mm anterior, −3.0 mm lateral, and 1.8 mm vertical) after drilling a port through the calvaria. A dummy cannula was inserted into the guide cannula, and allowed to recover for at least 5 days. A microdialysis probe (Eicom) was inserted into hippocampal region via the guide cannula. The probe was perfused with a Ringer’s solution, containing 147 mM NaCl, 3.0 mM KCl, 1.2 mM CaCl_2_, and 1.2 mM MgCl_2_, at a flow rate of 1.0 μL/min. MHBA were administered orally at a dose of 10 mg/kg. Microdialysis samples were collected every 20 min before and after administration. NE was quantified using HPLC-ECD (Eicom).

### Y-maze test

The effect of MHBA on spatial working memory was evaluated using the Y-maze test as described previously^29^, with slight modifications. The experimental apparatus used in this study consisted of three black arms, each of which was 250 mm long, 200 mm high, and 50 mm wide, positioned at equal angles. This apparatus was placed in a sound-isolated experimental room and observed through digital video camera mounted on the ceiling above the Y-maze. Mice were moved into this room at 16 h before the test. Test compounds were administered orally at 60 min before the test. Scopolamine (0.8 mg/kg), a muscarinic antagonist, was administered intraperitoneally at 20 min before the test, in order to induce temporal amnesia. In the β-AR inhibition test, propranolol (10 mg/kg) and scopolamine were intraperitoneally administered 20 min before the test. Each mouse was placed at the end of an arm and allowed to move freely in the Y-maze for 8 min. Spontaneous alternation behavior was defined as the successive entry of mice into each of the three arms in overlapping triplet sets. Spontaneous alternation was calculated using the following formula: (100 × number of spontaneous alternation behaviors)/(total number of arm entries-2).

### Novel object recognition test (NORT)

The effect of MHBA on episodic memory was evaluated using the novel object recognition test (NORT). The NORT was performed as described previously^35^. The experimental apparatus used in this study was a square open field (40 cm × 40 cm × 40 cm) made of grey polyvinyl chloride. The box was placed in a sound-isolated experimental room. Two pairs of wooden blocks were used as objects: two triangle prisms and two square pyramids. Each object was placed in a corner on the same side as the matching shape. The NORT was comprised of two periods: an acquisition period and a recall period. Mice were moved into the experimental room for at least 16 h prior to the acquisition period. Each mouse was then placed into the experimental apparatus in the presence of two objects, and allowed to explore freely for 10 min. After 24 h, the recall period was performed. During the recall period, one object of each pair was replaced with a novel object (wooden white sphere). Each mouse was again placed into the apparatus for 5 min, and the exploration time with the familiar object and novel object was measured. Test compounds were administered orally at 60 min before the acquisition period and recall period, respectively. The discrimination index was calculated using the following formula: (novel object exploration time minus familiar object exploration time)/(total exploration time).

### Statistical analysis

All values are expressed as means ± SEM. Two-group comparisons were analyzed by student’s *t* test. All other experimental data were analyzed by one-way ANOVA, followed by Dunnett’s test or Tukey-Kramer’s test. P < 0.05 was considered statistically significant.

## Electronic supplementary material

Supplementary information
