# Supplementary material for: Matured Hop-Derived Bitter Components in Beer Improve Hippocampus-Dependent Memory Through Activation of the Vagus Nerve
Source: Sci Rep. 2018 Oct 18;8:15372. doi: 10.1038/s41598-018-33866-1 (PMC6194057; doi:10.1038/s41598-018-33866-1)
Supplement: Supplementary file 1 — Supplementary information [file 41598_2018_33866_MOESM1_ESM.pdf]

Supplementary Information

Matured Hop-Derived Bitter Components in Beer Improve Hippocampus-Dependent Memory Through Activation of the Vagus Nerve

Tatsuhiro Ayabe*[1], Rena Ohya[1], Yoshimasa Taniguchi[2], Kazutoshi Shindo[3], Keiji Kondo[1], and Yasuhisa Ano[1].

1 Research Laboratories for Health Science & Food Technologies, Kirin Company Ltd., Yokohama, Kanagawa, 236-0004, Japan

2 Central Laboratories for Key Technologies, Kirin Company Ltd., Yokohama, Kanagawa, 236-0004, Japan

3 Department of Food and Nutrition, Japan Women's University, Tokyo, 112-8681, Japan

Supplementary Experimental Procedures

Assessment of vagotomy

Vagotomy was assessed by food intake analysis based on the satiety effect of cholecystokinin-octapeptide (CCK-8; Sigma Aldrich, St.Louis, MO, USA). Satiety effect of CCK-8 is mediated by afferent vagus nerves. At 16 h of food deprivation, sham and vagotomized mice were injected with 8 μg/kg CCK-8 or saline intraperitoneally. Food intake was then monitored for 30 min.

Preparation and intracerebroventricular injection of amyloid β oligomer

Amyloid β oligomer (Aβ1-42) was dissolved in hexafluoro-2-propanol (HFIP, Wako) to 1 mM and incubated at room temperature for 30 min. HFIP was removed by volatilization and Aβ peptide film was obtained. Aβ was then dissolved in dimethyl sulfoxide to 5 mM and resuspended in PBS to 100 μM. This solution was incubated at 4 °C for 24 hrs to induce aggregation of Aβ. The solution was centrifuged at 10,000 rpm for 15 min and the supernatant was derived as Aβ oligomer solution. Mice were anesthetized using pentobarbital (Somnopentyl; Kyoritsu, Tokyo, Japan). 100 μM Aβ oligomer or PBS was injected intraventicularly at 5 μL for both hemispheres. Mice were allowed for 3 days to recover and used for the Y-maze test.

Preparation of 4′-hydroxy-*cis*-alloisohumulone

4′-Hydroxy-*cis*-alloisohumulone (HAIH) was purified from MHBA. The MHBA were subjected to reversed-phase preparative HPLC [column: 150 × 22 mm id, 5 μm, Alltima C_{18} column (Systech, Tokyo, Japan); solvent: H_2O/H_3PO_4 (85%), 100/1, (v/v) (solvent A) and MeCN (solvent B), a linear gradient from 30 to 75% B in 0 → 30 min, 75 to 90% B in 30 → 30.1 min and 90% B for 30.1 → 37 min; flow rate: 22.8 mL/min]. The fraction containing HAIH was further purified by 2nd preparative HPLC [column: 150 × 20 mm id, 5 μm, L-column 2 ODS (Chemicals Evaluation and Research Institute, Tokyo, Japan); solvent: 100 mM NH_4HCO_3 (solvent A) and MeCN (solvent B), isocratic elution at 25% B; flow rate: 9.5 mL/min], yielding pure HAIH.

4′-Hydroxy-cis-alloisohumulone (HAIH): pale yellow oil; $[\alpha]^{20}_{D}$ +76.2 (*c* 0.3, MeOH); HRESIMS (negative) *m/z* 377.1965 $[M - H]^-$ (calcd for $C_{21}H_{29}O_6$, 377.1970); ^1H NMR (400 MHz, methanol-*d₄*) and ^{13}C NMR (100 MHz, methanol-d_4) spectra were identical to the literature data[1] , and are given in Supplementary Table 1.

Reference

1. Intelmann D, Hofmann T. On the autoxidation of bitter-tasting iso-alpha-acids in

beer. *Journal of agricultural and food chemistry* 2010; **58**(8)**:** 5059-5067.

Supplementary Table 1. NMR spectroscopic data (400 MHz, methanol-d_4) for 4'-hydroxy-*cis*-alloisohumulone

4'-hydroxy-*cis*-alloisohumulone		
pos.	δ_C, type	δ_H, mult. (J in Hz)
1	205.2, C	-
2	112.5, C	-
3	198.3, C	-
4	87.8, C	-
5	52.7, CH	3.14, dd (7.1, 5.9)
1'	199.5, C	-
2'	120.5, CH	6.85, d (15.6)
3'	157.2, CH	7.02, d (15.6)
4'	71.5, C	-
5'	29.1, CH_3	1.31, s
6'	29.2, CH_3	1.31, s
1"	26.2, CH_2	a: 2.43, m<br>b: 2.38, m
2"	121.8, CH	5.10, m
3"	135.1, C	-
4"	17.8, CH_3	1.59, s
5"	26.0, CH_3	1.62, s
1'''	200.6, C	-
2'''	47.2, CH_2	a: 2.74, dd (13.9, 7.1)<br>b: 2.69, dd (13.9, 7.0)
3'''	27.2, CH	2.10, m
4'''	22.8, CH_3	0.94, d (6.6)
5'''	22.9, CH_3	0.95, d (6.6)

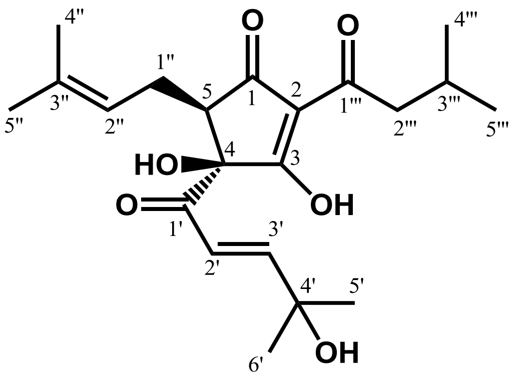

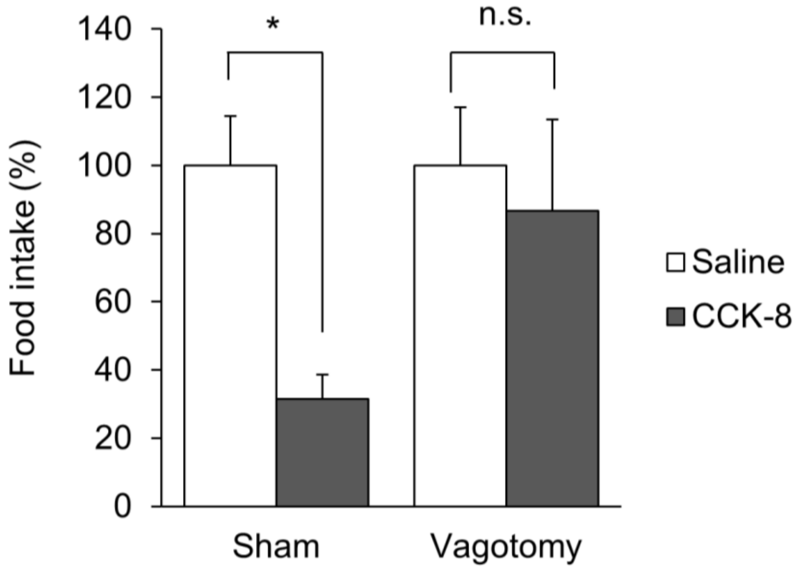

Supplementary Fig. 1. Satiety effect of CCK-8 is not observed in vagotomized mice

Vagotomy was assessed by analyzing food intake after CCK-8 treatment. Mice were first injected with CCK-8 (8 μg/kg) intraperitoneally and food intake was monitored for 30 min. Two days later, mice were then injected with saline and food intake was monitored. Food intake after CCK-8 treatment was expressed as a percentage of that of saline treated mice. *p<0.05 versus each saline treated group.

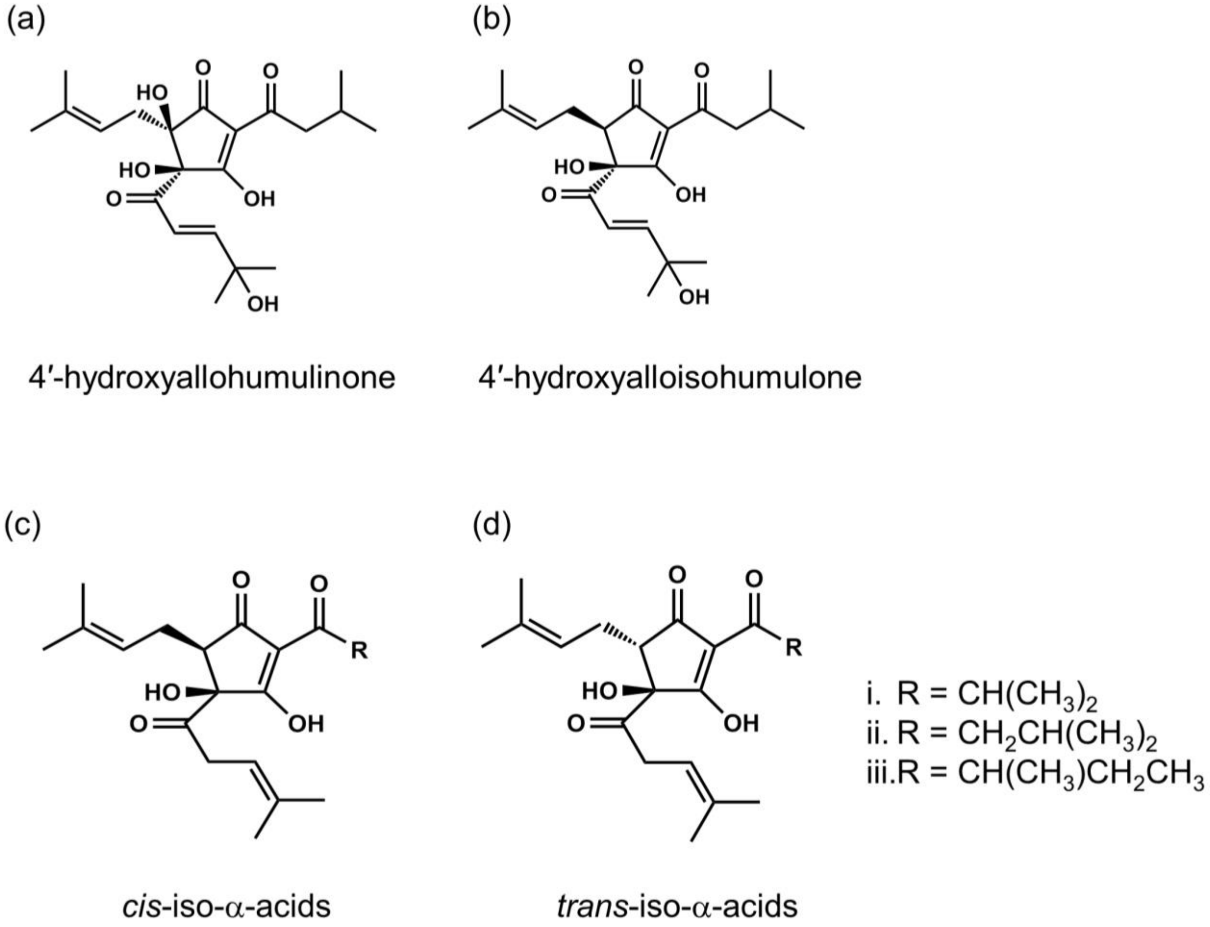

Supplementary Fig. 2. Chemical structures of HAH, HAIH, and iso-α-acids

(a) 4′-hydroxyallohumulinone (HAH). (b) 4′-hydroxyallo-*cis*-isohumulone (HAIH). (c) *cis*-iso-α-acids, *cis*-isocohumulone (i), *cis*-isohumulone (ii), and *cis*-isoadhumulone (iii). (d) *trans*-iso-α-acids, *trans*-isocohumulone (i), *trans*-isohumulone (ii), and *trans*-isoadhumulone (iii).

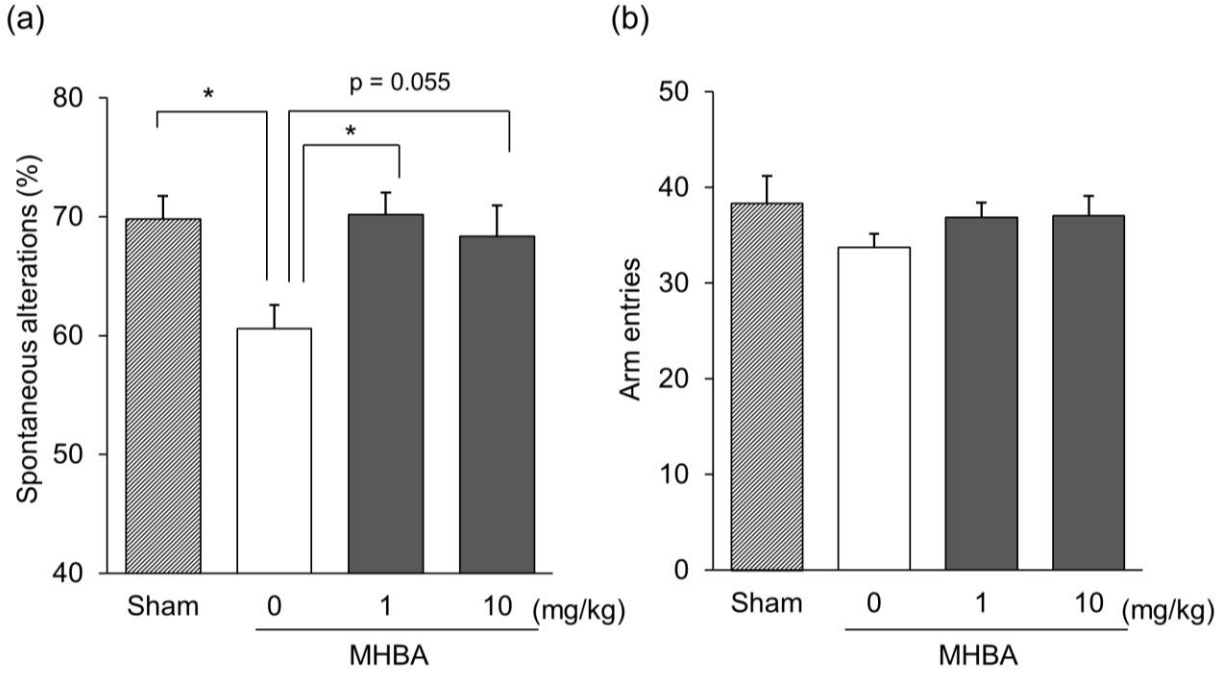

Supplementary Fig. 3. MHBA improve spatial working memory in amyloid β oligomer-treated amnesia mice

Mice were injected with Aβ oligomer intraventricular and amnesia was induced at 3 days before the Y-maze test. MHBA (0, 1, 10 mg/kg) were administered orally. At 60 min after the oral administration, spontaneous alteration behavior (a) and number of arm entries (b) in the Y-maze was observed for 8 min. All values are expressed as means ± SEM (n=14-16 mice per group). *p<0.05 versus each group.
